# Infrared Thermographic Assessment of Cooling Effectiveness in Selected Dental Implant Systems

**DOI:** 10.1155/2016/1879468

**Published:** 2016-03-15

**Authors:** Karol Kirstein, Maciej Dobrzyński, Piotr Kosior, Aleksander Chrószcz, Krzysztof Dudek, Katarzyna Fita, Olga Parulska, Zbigniew Rybak, Aleksandra Skalec, Magdalena Szklarz, Maciej Janeczek

**Affiliations:** ^1^Department of Biostructure and Animal Physiology, Wroclaw University of Environmental and Life Sciences, Kożuchowska 1, 51-631 Wroclaw, Poland; ^2^Department of Conservative Dentistry and Pedodontics, Wroclaw Medical University, Krakowska 26, 50-425 Wroclaw, Poland; ^3^Faculty of Mechanical Engineering, Technical University of Wroclaw, Łukasiewicza 5, 50-371 Wroclaw, Poland; ^4^Department of Dental Surgery, Wroclaw Medical University, Krakowska 26, 50-425 Wroclaw, Poland; ^5^Department of Experimental Surgery and Biomaterial Research, Wroclaw Medical University, Poniatowskiego 2, 50-326 Wroclaw, Poland

## Abstract

The excessive temperature fluctuations during dental implant site preparation may affect the process of bone-implant osseointegration. In the presented studies, we aimed to assess the quality of cooling during the use of 3 different dental implant systems (BEGO®, NEO BIOTECH®, and BIOMET 3i®). The swine rib was chosen as a study model. The preparation of dental implant site was performed with the use of 3 different speeds of rotation (800, 1,200, and 1,500 rpm) and three types of cooling: with saline solution at room temperature, with saline solution cooled down to 3°C, and without cooling. A statistically significant difference in temperature fluctuations was observed between BEGO and NEO BIOTECH dental systems when cooling with saline solution at 3°C was used (22.3°C versus 21.8°C). In case of all three evaluated dental implant systems, the highest temperature fluctuations occurred when pilot drills were used for implant site preparation. The critical temperature, defined in the available literature, was exceeded only in case of pilot drills (of all 3 systems) used at rotation speed of 1,500 rpm without cooling.

## 1. Introduction

Dental implants-related topics are an important matter of contemporary human and veterinary dentistry. During placement of dental implants into the bones of the facial skeleton, different cooling systems are used [1–3]. They provide maintenance of an appropriate temperature and prevent tissue overheating during an exothermic dental implant insertion procedure [3–5].* In vivo* temperature measurements can be performed using thermocouple or thermography. Our work tries to use the drilling tool as a “thermosensor” analyzed with infrared thermography. Thermographic method allows the noninvasive observation of bone drilling process. Thermographic camera showed the temperature changes in the external surface of bone and visible part of the drilling tool. Moreover, the whole surface of drilling was investigated directly after the bone canal creation. Infrared thermography is noninvasive and completely safe for the patient method that can be used to calculate heat emission during drilling the hole in the bone [6, 7]. As a result, we have selected the infrared thermographic analysis to determine the most effective cooling system. A swine rib is a commonly used animal model that is comparable to a large extent with the structure of human mandible [3]. Infrared thermography is a method that is commonly used in experimental studies to assess thermal changes in tissues and organs of animals. Therefore, its use in this paper is justified [8–12]. The paper aimed at determining which of the dental implant site preparation systems has better cooling parameters and does not induce adverse changes in the bone tissue.

## 2. Material and Methods

In this paper, we used 9 fresh swine ribs taken from great white (Polish) pigs, designated by consecutive Arabic numerals (1–9). The length, width, and thickness of all ribs were similar and comparable; the mean values of above-mentioned parameters were 147,2 mm × 24,6 mm × 21,3 mm. Three following dental implant systems were investigated (Table 1):

(i) BEGO (drills: pilot, *ϕ*2.5, and *ϕ*3.0).
(ii) BIOMET 3i (drills: pilot, *ϕ*2.3, and *ϕ*2.75).
(iii) NEO BIOTECH (drills: pilot, *ϕ*2.2, and *ϕ*2.9).

Each of the above-mentioned dental implant systems was tested under the following parameters:

- Cooling:
  - external cooling with 0.9% NaCl solution stored at room temperature (app. 20°C),
  - external cooling with 0.9% NaCl solution (so-called cold saline) stored at temperature app. 3°C,
  - without cooling.
- Drill rotation speed:
  - 800 rpm,
  - 1,200 rpm,
  - 1,500 rpm.

In the study, we used a dental implant micromotor NeoSurge (NEO BIOTECH) equipped by the manufacturer with a contra angle with 32 : 1 gear reduction. Each subsequent dental implant site preparation was performed on the external surface of the rib by perforating the lamina of the compact substance and reaching diploe, with maintaining constant preparation depth (10 mm). Each bone fragment was mounted on a stable working stand; the drilling procedure was performed by the same experienced surgeon with the use of an optimal contact force. The drilling procedure was registered with the use of infrared thermographic camera ThermaCAM P640 (FLIR) with a spectral range of 7.5–13 *μ*m and matrix of 640 × 480 pixels. Thermograms registered at the frequency of 30.15 Hz were analyzed in order to determine the maximal temperature within the region of interest (ROI) involving bone and drill. The measurement system enabled calculation of drilling time and temperature change within the region of interest (ROI). In this study, each drill was used only three times (at 800, 1,200, and 1,500 rpm), which significantly decreases the possibility to blunt the drill and to generate additional heat. The measurements were performed in a closed room under equal conditions of temperature and humidity and without air flow. The drilling procedures were performed by an experienced implantologist, which ensured repeatability of tests results.

The statistical analysis was conducted with the use of STATISTICA (StatSoft, Inc., Tulsa, USA) software.

## 3. Results

**Figure 1:**
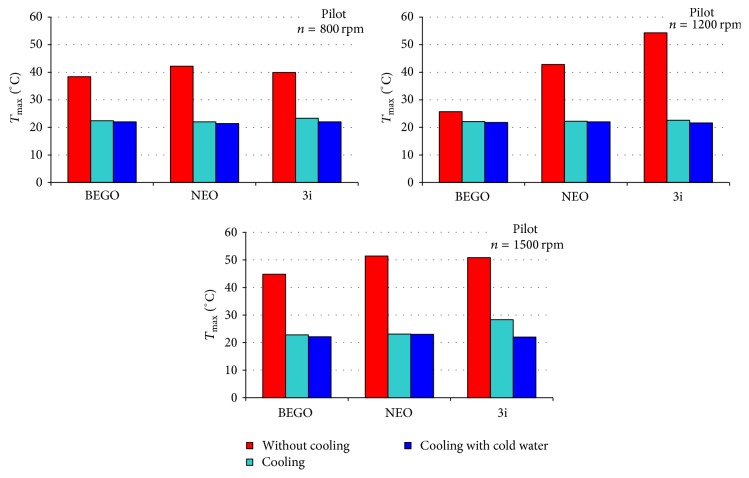
Comparison of maximal temperatures recorded during drilling of holes in the bone fragment with the use of pilot drill.

**Figure 2 fig2:**
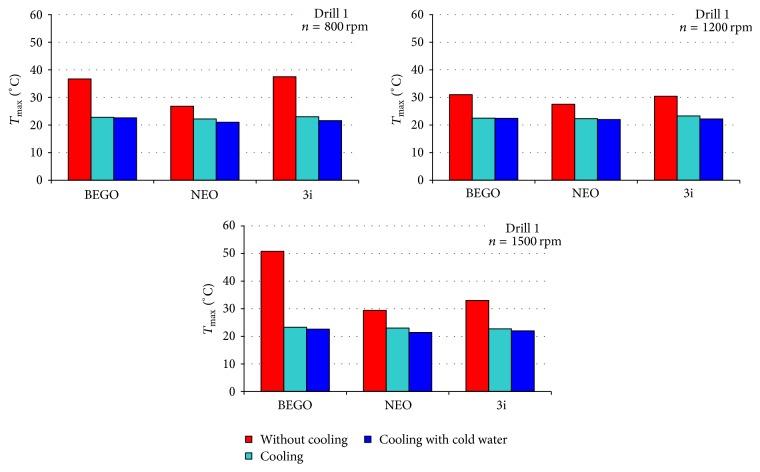
Comparison of maximal temperatures recorded during drilling of holes in the bone fragment with the use of intermediate drill.

Table 2 presents temperatures obtained during drilling performed at specified time point, with or without cooling system. Figures 1–3 present comparison of infrared thermographic assessments with regard to rotation speed of three subsequent drills used in three selected dental implant systems. Figures 4-5 present results of statistical analysis. A statistically significant difference in temperatures was observed only between BEGO and NEO BIOTECH dental systems when cooling with “cold” saline solution was used (22.3°C versus 21.8°C, *p* = 0.024) (Figure 4).

Without cooling, drilling time with NEO BIOTECH system is significantly shorter compared to BEGO (1.76 s versus 2.72 s, *p* = 0.035) and BIOMET 3i systems (1.76 s versus 3.25 s, *p* = 0.001). With cooling, drilling time with NEO BIOTECH system is significantly longer compared to BEGO (3.68 s versus 2.33 s, *p* = 0.005) and BIOMET 3i systems (3.68 s versus 2.70 s, *p* = 0.044). When cooling with “cold” saline is used, differences in drilling times are insignificant (*p* > 0.05) (Figure 5).

## 4. Discussion

The above-mentioned studies aimed at the assessment of the efficacy of cooling used in three different dental implant systems depending on different drill diameters and rotation speeds. It was proven that a wide range of different factors impact the heat emitted during dental implant site preparation. The above-mentioned factors involve cortical lamina thickness, rotation speed, drill diameter, drill geometry, and penetration depth [3, 13–17]. Previous study revealed that during dental implant site preparation a temperature exceeding 47°C negatively impacts bone-implant osseointegration. In addition, the above-mentioned study demonstrated that drilling time below 1 minute and temperature not exceeding 47°C positively impact procedure success [18]. On the other hand, studies by other authors showed that temperature above 50°C, accompanied by the prolongation of preparation time, is critical and induces thermal necrosis [3, 19]. In different dental implant systems, the optimal rotation speed that stimulates osseointegration and does not induce bone overheating ranges from 1,000 to 1,500 rpm [3]. In our studies, we used three different rotation speeds: suboptimal (800 rpm), optimal (1,200 rpm), and maximal (1,500 rpm). Our studies demonstrate that rotation speed of 800 rpm is associated with a contact force that generates more heat, while maximal rotation speed (1,500 rpm) is accompanied by an increased heat emission due to substantial increase of friction. In their studies, Chacon et al. [13] demonstrated an association between the amount of emitted heat and the degree of drill wear, which indicates the need of further trials involving the above-mentioned dental implant systems. It should be emphasized that dental implant micromotor working at low rotation speed allows surgeon to adjust drilling trajectory compared to manual technique [3]. Heat energy generated during drilling can be evaluated using thermocouple or thermography [3, 19]. The aim of this study was to evaluate the drilling parameters influence on temperature changes within the bone tissue. The amount of energy release depends on many factors (i.e., the bone and drill type as well as the method of drilling). During the bone drilling, the direct access to the bone surface, which is being drilled, is limited and the direct temperature measurement at the same time is impossible. The miniature contact sensors (e.g., thermoelements, thermoresistors, and thermistors) usage, despite their accuracy and electric signal elaboration simplicity, is not beneficial not from economical point of view only. The necessity of their allocation within the investigated bone tissue may also lead to structure changes in the tissue. Therefore, similar to other authors [3, 20, 21], we decided to use the noninvasive pyrometric method. The development of noninvasive temperature measurement tools, based on the heat energy emission (radiation) detection and quantification, allows the temperature areal evaluation during drilling. Therefore infrared thermography, as a noninvasive, repetitive, and relatively fast method, is particularly valuable option in quantitative assessment of the emitted heat generated in experimental models [7, 11], not to mention the fact that infrared thermographic camera alone enabled successful observation of quantitative changes of the heat emitted during the study [3, 20, 22–25].

The thermography determines temperature changes at the external surface of the bone and visible part of the drill. Taking under consideration the latter assumption, the preliminary studies were carried out in the same material (bone tissue) and consisted of secondary drilling within the primary 1 mm diameter perforation canals. The thermographic camera was located contralateral to the drill canal long axis. This camera-drill relation allows continuous observation of drill tip surface and its temperature measurement during the whole manipulation until the complete removal of tool from the drilled canal. The thermographic analysis proved that the difference between maximal temperature and the value recorded directly after drill evaluation from bone canal equals ca. 0,7°C ± 0,2°C. The achieved result was constant and it was taken into consideration during the main thermographic analysis.

**Figure 3:**
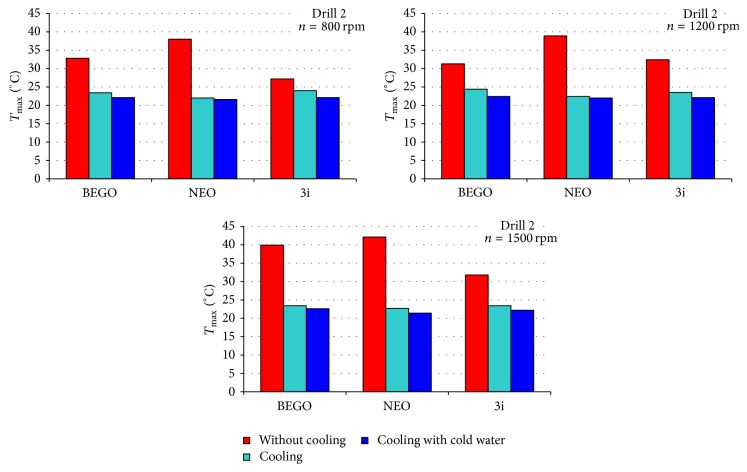
Comparison of maximal temperatures recorded during drilling of holes in the bone fragment with the use of final drill.

**Figure 4:**
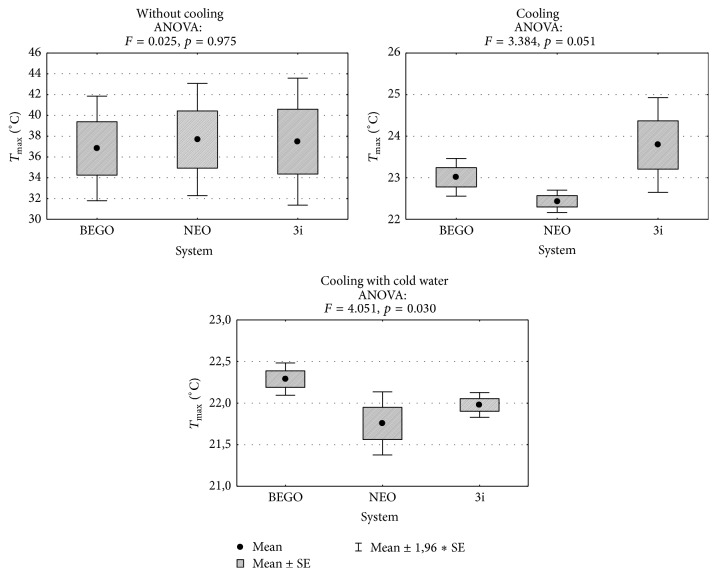
Comparison of maximal temperatures recorded during drilling procedure with the use of all three drills and results of the analysis of variance.

**Figure 5:**
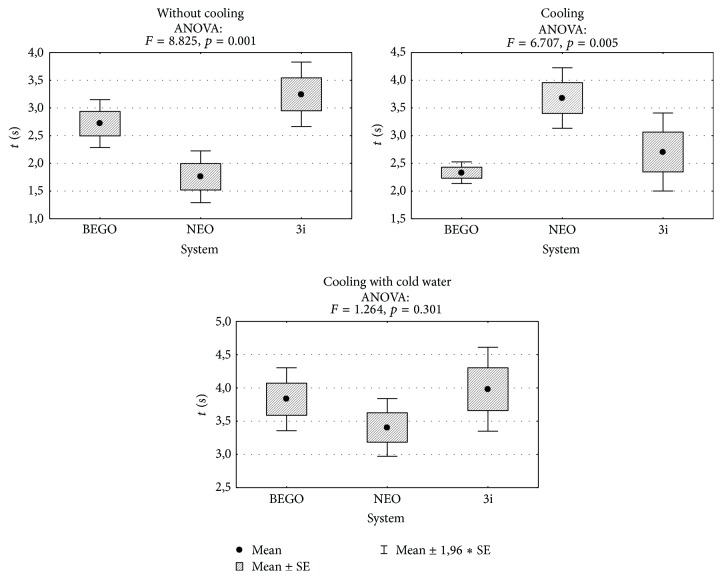
Comparison of drilling times for each of the examined dental implant systems and results of the analysis of variance.

It is well known that temperature measurement is affected by a wide range of factors, that is, room temperature, humidity, ventilation, and the presence of external heat sources [11]. As a result, in our study all stages of thermographic assessment were performed in the same environmental conditions. The use of infrared thermography enabled assessment of the maximal temperature for each dental implant system. Without cooling, the mean temperature generated by the evaluated systems (with the use of 2.2–2.5 mm drills and rotation speed of 1,200 rpm) was 27–31°C. Kim et al. [3], in turns, evaluated Brånemark®, Osstem®, and Bicon® systems and received higher mean maximal temperature that without cooling was fluctuating between 32°C and 34°C. In the above-mentioned studies, 2-3 mm drills and analogical rotation speed were used. In accordance with many previous reports, the use of appropriate cooling systems, apart from drill diameter and rotation speed, is an important factor reducing the risk of osteonecrosis [21, 26, 27]. During implantation the external or internal cooling techniques can be used. The lack of internal cooling system analysis in our study is caused by chosen implantation system construction. Majority of modern implantation systems do not include internal cooling because of drill sterilization problems (higher infection risk) and greater bone tissue loss (water erosion). Moreover the clinical studies of implant osteointegration with a bone proved the lack of significant differences between two investigated implantation systems (with external or internal cooling). The pilot drill usually is not equipped with internal cooling system, despite the main one using the system. The thermographic analysis confirmed an important impact of cooling on reduction of the temperature generated by a dental implant system during drilling procedure (Figures 1–3). In addition, our analyses aimed to determine a potential impact of coolant's temperature reduction on the quality of cooling procedure. However, our results clearly demonstrate that minimal differences in temperature are not statistically significant (Figures 4 and 5). Therefore, the use of the above-mentioned liquid stored at room temperature and at 3°C does not significantly improve the efficacy of cooling (Figures 1–3). The cooling process alone, used in different dental implant systems, may impact the preparation time, for example, by increasing (e.g., NEO system) or decreasing (e.g., two other systems used in this study) the drilling time. The analysis of the above-mentioned dental implant systems has proved that there are differences in drilling time between those systems (when used with optimal rotation speed [1,200 rpm] and drills of an intermediate diameter). The analysis of our results suggests that the use of a pilot drill at all three rotation speeds selected for the purpose of this study results in approximation towards or exceeding the critical temperature; therefore, it absolutely requires the use of a cooling system. The comparison of instantaneous increases of temperature indicates that an irreversible damage of bone tissue is more often induced during preparation of the pilot hole compared to proper dental implant site.

## 5. Conclusions

1. During dental implant site preparation, temperature fluctuations are directly related with the use of cooling system, drill diameter, and rotation speed of the micromotor.
2. No important difference between the coolant's temperature and temperature fluctuations within the implant site was observed.
3. In all three systems used in this study, important temperature fluctuations were observed during implant site preparation with the use of pilot drills.
4. The NEO BIOTECH system is characterized by the shortest time of implant site preparation.
5. The critical temperature, defined in the available literature, was exceeded only in case of pilot drills (of all 3 systems) used at rotation speed of 1,500 rpm without cooling.

## Figures and Tables

**Table 1:** Evaluated drilling systems variants.

Number	Rib	System	Comments
1	Number 1	BEGO Implant Systems	Without cooling
2	Number 2	BEGO Implant Systems	With cooling
3	Number 3	BEGO Implant Systems	Cooling with cold saline
4	Number 4	NEO BIOTECH	Without cooling
5	Number 5	NEO BIOTECH	With cooling
6	Number 6	NEO BIOTECH	Cooling with cold saline
7	Number 7	BIOMET 3i	Without cooling
8	Number 8	BIOMET 3i	With cooling
9	Number 9	BIOMET 3i	Cooling with cold saline

**Table 2:** Maximal temperature within ROI.

Drill	System	*n* (rpm)	Without cooling		Cooling		Cooling with cold saline	
			*t* [s]	*T*_max_ (°C)	*t* [s]	*T*_max_ (°C)	*t* [s]	*T*_max_ (°C)
Pilot	BEGO	800	2.82	38.4	2.49	22.4	2.85	22.0
Pilot	BEGO	1,200	1.96	25.7	2.16	22.1	3.25	21.8
Pilot	BEGO	1,500	3.58	44.8	2.69	22.8	3.32	22.1
*ϕ*2.5	BEGO	800	3.42	36.7	2.52	22.8	5.27	22.6
*ϕ*2.5	BEGO	1,200	3.45	31.0	2.72	22.5	3.95	22.4
*ϕ*2.5	BEGO	1,500	2.89	50.8	2.16	23.3	3.78	22.6
*ϕ*3.0	BEGO	800	1.99	32.8	2.23	23.4	4.48	22.1
*ϕ*3.0	BEGO	1,200	2.19	31.3	1.79	24.4	3.55	22.4
*ϕ*3.0	BEGO	1,500	2.16	39.9	2.23	23.4	4.01	22.6
Pilot	NEO	800	3.05	42.2	3.58	22.0	3.12	21.4
Pilot	NEO	1,200	2.69	42.8	3.45	22.2	3.98	22.0
Pilot	NEO	1,500	1.92	51.4	4.91	23.1	3.15	23.0
*ϕ*2.2	NEO	800	1.56	26.8	3.58	22.2	3.35	21.0
*ϕ*2.2	NEO	1,200	1.29	27.5	3.85	22.3	3.52	22.0
*ϕ*2.2	NEO	1,500	1.06	29.4	3.32	23.0	3.35	21.4
*ϕ*2.9	NEO	800	1.03	38.0	2.79	22.0	2.99	21.6
*ϕ*2.9	NEO	1,200	1.29	38.9	5.04	22.4	4.78	22.0
*ϕ*2.9	NEO	1500	1.92	42.1	2.59	22.7	2.42	21.4
Pilot	3i	800	3.28	39.9	5.21	23.3	4.15	22.0
Pilot	3i	1,200	4.74	54.3	3.18	22.6	2.49	21.6
Pilot	3i	1,500	3.88	50.8	3.12	28.3	3.42	22.0
*ϕ*2.3	3i	800	3.75	37.5	2.45	23.0	5.74	21.6
*ϕ*2.3	3i	1,200	3.65	30.4	1.89	23.3	3.98	22.2
*ϕ*2.3	3i	1,500	2.79	33.0	2.26	22.7	5.04	22.0
*ϕ*2.75	3i	800	1.79	27.2	2.36	24.0	4.01	22.1
*ϕ*2.75	3i	1,200	2.29	32.4	1.49	23.5	3.18	22.1
*ϕ*2.75	3i	1,500	3.05	31.8	2.39	23.4	3.81	22.2
